# Epidemiological characteristics of Pandemic Influenza A (H1N1-2009) in Zhanjiang, China

**Published:** 2011-12-14

**Authors:** JinJian Fu, SiDong Chen, JiaLin Chen, Jie Wang, ChenWen Ling

**Affiliations:** 1 Department of Epidemiology, Southern Medical University, Guangzhou, China; 2 Influenza Laboratory, Center for Disease Control and Prevention of Zhanjiang, Zhanjiang, China; 3 School of public health, Guangdong Pharmaceutical University, Guangzhou, China

**Keywords:** Disease outbreaks, epidemiology, Influenza A, H1N1, China

## Abstract

**Background:**

A novel influenza A virus strain (H1N1-2009) spread first in Mexico and the United Stated in late April 2009, leading to the first influenza pandemic of the 21^st^ century. The objective of this study was to determine the epidemiological and virological characteristics of the pandemic influenza A (H1N1-2009) in Zhanjiang, China.

**Methods:**

The case and outbreak reports of influenza-like illness (ILI) were collected from the Chinese information system of disease control and prevention and the influenza surveillance system of Zhanjiang city. Real-time RT-PCR was conducted, and epidemic and virological characteristics of the virus were analyzed using descriptive epidemiological methods and Chi-square trend tests.

**Results:**

A total of 276 reported cases were confirmed from July 16, 2009 to June 30, 2010. The attack rate of outbreak was from 1.1% to 6.0%. The disease peak occurred in December 2009, after which the outbreak subsided gradually. The last case was confirmed in April 2010.

**Conclusion:**

The main population struck by the H1N1-2009 virus was young adults, youths and children. The outbreaks most frequently occurred in schools, and most cases were acquired locally

## Background

In late April 2009, a novel influenza A (H1N1-2009) virus strain spread in Mexico and the United States [1,2]. Over the next two months, the virus spread rapidly around the world, resulting in the declaration of the first influenza pandemic of the 21^st^ century on June 11, 2009 by the World Health Organization (WHO) [3]. Influenza is the only globally monitored infectious disease, and Centers for Disease Control and Prevention of China has established a nationwide network of influenza monitoring system. The influenza laboratory of Center for Disease Control and Prevention of Zhanjiang (Zhanjiang CDC), one of the earliest eight influenza laboratories established in Guangdong Province in 1998, joined the national influenza monitoring network in June 2009, and was designated as the only approved laboratory for diagnosing pandemic influenza A (H1N1-2009) virus infection in Zhanjiang. This article describes the epidemiological and virological aspects of the H1N1-2009 influenza A cases reported from sentinel surveillance and outbreaks.

## Methods

### Epidemic evaluations

Based on the “Guidelines for Pandemic Influenza A (H1N1-2009)” published by the Ministry of Health of China on April 30, 2009 [4], the Guangdong Bureau of Health has activated a series of pandemic preparedness plans. Since June 2009, Guangdong province has implemented an expanding pandemic monitoring project. Various indicators of influenza activities were monitored according to this project, including (i) percentage of visit for influenza-like illness (ILI%), (ii) percentage of visit for pandemic influenza A (H1N1-2009) (PI%), and (iii) percentage of visit for seasonal influenza (SI%). The percentages were determined with formulas ILI% = D/E, PI% = (A/C)×(D/E), and SI% = (B/C) × (D/E), in which A is the number of seasonal influenza-positive cases detected by the influenza etiological monitoring laboratory, B is the number of confirmed H1N1-2009 cases, C is the number of nasopharyngeal swab specimens collected from sentinel hospitals, D is the number of influenza-like illness received from sentinel hospitals, and E is the number of patients who visited the sentinel hospitals.

### Definition of infection

Influenza-like illness was defined as a fever of more than 37.5°C with one or more signs or symptoms, including sore throat, cough, runny nose, and nasal congestion. A confirmed case was defined as a laboratory confirmation of infection by positive PCR results obtained from specimen on a nasopharyngeal swab.

### Sample collection

Each week, nasopharyngeal swabs were obtained from 20–30 ILI patients of various age groups in sentinel hospitals and shipped to CDC in viral transport media. If there was an outbreak, nasopharyngeal swabs obtained from at least 10 patients with suspected cases were sent to the Zhanjiang CDC for the virus detection. Epidemiologic and demographic parameters were obtained for each patient.

### Detection of virus infection

Real-time reverse transcription-PCRs (RT-PCR) specific for matrix gene sequences of influenza A and B viruses [5] were used to diagnose influenza virus infections and to determine specific subtypes. RNA was extracted using the QIAamp Viral RNA Mini Kit (QIAGEN, Valencia, CA, USA). All suspected cases were required to be reported, and the nasopharyngeal swab specimens were shipped to Zhanjiang CDC within 24 hours of diagnosis. Statistical analyses were performed using X^2^ tests.

## Results

### Epidemic of influenza A (H1N1-2009) in Zhanjiang from June 2009 to June 2010

Influenza reinforcement monitoring has been carried out from May 2009 to February 2010 in Zhanjiang. The first H1N1-2009 case (also the first known imported case) was confirmed in Zhanjiang on July 16, 2009. By the end of June 15, 2010, a total of 276 H1N1-2009 cases have been reported. Among these, six were severe cases (represented about 2.2% of H1N1-2009 cases), which all recovered and no death was reported. The rest of these cases arose from local transmission except for the 37 imported cases. The highest recorded ILI rate occurred in E-week 38 (September 13–18). Epidemiological investigations indicated that the peak wave of H1N1-2009 epidemic occurred in December, in which 94 cases were detected. Then the number of confirmed cases dropped sharply with only some sporadic occurrence, and the last case was detected on April 27, 2010.

### Epidemiological characteristics of pandemic influenza A (H1N1-2009)

The first imported case confirmed on July 16, 2009 was detected from a group of 7- to 12-year-old male pupils, who were on a summer camp trip in Guangzhou from July 5 to 10, and returned to Zhanjiang on July 11. One of them had a fever the next morning. A total of 13 out of 25 campers developed ILI from July 12 to 15. The situation was reported to Zhanjiang Bureau of Health on July 16. Nasopharyngeal swabs were collected from 13 male suspected cases, and seven were tested positive for H1N1-2009 virus by RT-PCR.

The first locally infected case of H1N1-2009 was reported on August 12, 2009 from routine general sentinel surveillance. This case was a 7-year-old girl who became ill on August 7 and was sent to the hospital on August 11. The girl had no travel history or contacts with travelers. A nasopharyngeal swab was collected from the patient and she was also tested positive for H1N1-2009.

Among the 1302 samples collected from July 2009 to June 2010, 276 (21.2%) were tested positive for the H1N1-2009 virus using RT-PCR. Among these 276 patients, 167 (60.5%) were male. The ages of the patients with confirmed infections ranged from 5 months to 59 years old, and the median age was 14. H1N1-2009 virus was detected most frequently among patients 6-15 years of age (42.8%), followed by those 16-30 years of age (39.9%). No confirmed case was detected in patients above 60 years old from routine general practice sentinel surveillance (Table 1).

**Table 1:** Patients with pandemic influenza A (H1N1-2009) in different age groups diagnosed at the influenza laboratory of Zhanjiang CDC from July 2009 to June 2010

Patient age (year)	Number of cases tested	Number of positive cases (%)	OR	P value	95% CI
0-5	544	44 (8.1)	0.74	0.412	0.34-1.63
6-15	493	164 (33.3)	4.19	0.000	2.04-8.82
16-30	171	58 (33.9)	4.31	0.000	1.99-9.57
> 30	94	10 (10.6)	1	-	-
Total	1302	276 (21.2)	-	-	-

### Pandemic influenza A (H1N1-2009) monitoring

**Figure 1:**
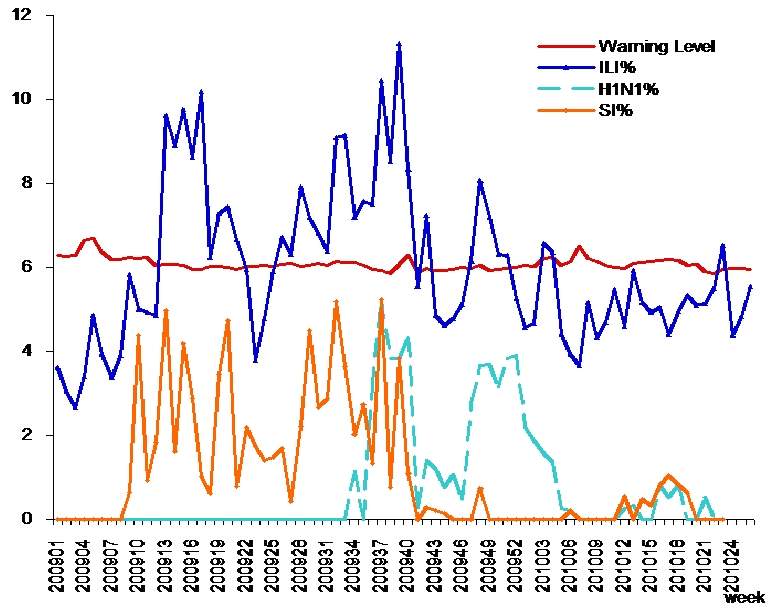
Weekly sentinel surveillance of influenza activities in Zhanjiang City from January 2009 to June 2010. ILI%: percentage of influenza-like illness; PI%: percentage of pandemic influenza A (H1N1-2009); SI%: percentage of seasonal influenza (SI %)

Figure 1 shows the weekly epidemiological data for influenza activities in Zhanjiang based on sentinel surveillance of ILI. Three peaks were seen in the curve of ILI%. The number of ILI cases exceeded the warning level during E-week 13-17 (March 22-April 25) when 26 patients were tested positive for seasonal influenza. The highest recorded ILI rate occurred at E-week 37-39 (September 6-26), which was caused by the co-circulation of seasonal influenza subtype A (H1N1, H3N2) and pandemic influenza A (H1N1-2009). The third peak was at E-week 48 (November 22-28), when 43 H1N1-2009 cases were confirmed. Sporadic cases had occurred during the summer vacation, and with the beginning of a new semester, influenza A (H1N1-2009) and seasonal influenza A (H3N2) viruses spread among schools with 23 separate outbreaks reported to the Zhanjiang CDC. Among these, 11 outbreaks were caused by seasonal influenza A (H3N2) virus, 11 outbreaks were caused by influenza A (H1N1-2009) virus, and one outbreak was caused by the co-circulation of influenza A (H1N1) and seasonal influenza subtype A (H3N2) viruses.

**Figure 2:**
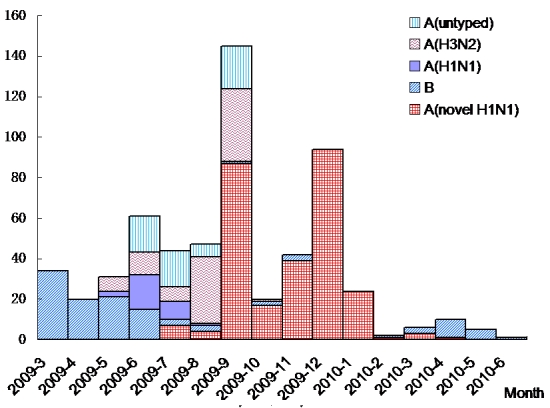
Comparison of influenza diagnostic testing results for specimens received in Zhanjiang City from March 2009 to June 2010

The Zhanjiang CDC assisted with the control of 11 outbreaks of H1N1-2009 virus in 11 separate schools in December. Influenza A (H1N1-2009) virus strain predominated at this time (Figure 2); 94 nasopharyngeal swab samples from outbreaks and routine general practice sentinel surveillance were ascertained for the presence of influenza A (H1N1) virus, which indicated the peak arrival of the pandemic influenza A (H1N1-2009).

### Influenza etiological monitoring

Figure 2 shows the outcome of influenza etiology obtained from all the nasopharyngeal swabs collected from March 2009 to June 2010. Influenza screening started in May 2009 after the Ministry of Health of China published the guidelines about surveillance, reporting, diagnosis, and treatment of pandemic influenza A (H1N1-2009) [4]. Influenza reinforcement monitoring started on June 23, 2009, when an expanding pandemic monitoring project was implemented in Guangdong province. Influenza viruses were isolated directly from the nasopharyngeal swabs received from sentinel hospital before June 23, 2009, and no PCR was performed on these nasopharyngeal swabs.

Zhanjiang experienced seasonal influenza and H1N1-2009 virus epidemics in 2009. Since March 2009, seasonal influenza strains had spread widely in Zhanjiang. The seasonal influenza B strain predominated in March, April and May, and seasonal influenza A dominated in June, July and August. In September, there was a rapid rise in the number of notifications of H1N1-2009 cases. Then the pandemic influenza A (H1N1-2009) virus dominated, and no seasonal influenza was circulating in the population. Since January 2010, the ILI rate had steadily decreased. The number of nasopharyngeal swabs received for testing had dropped about 50% and the number of confirmed H1N1 cases had dropped sharply. Then the outbreak passed through the peak and gradually subsided, with only sporadic seasonal influenza B and H1N1-2009 cases detected through sentinel surveillance. The last case of influenza A (H1N1-2009) was detected on April 27, 2010.

### Response strategies implemented in different phases of influenza pandemic

Since March, high infection and mortality rates have been reported in Mexico, which led to the declaration by the WHO the start of pandemic on June 11, 2009 [3]. The first imported case (also the first case) of pandemic influenza A (H1N1-2009) was confirmed in China on May 11, 2009 [6]. One week later, the first imported case was reported in Guangdong Province [7]. A variety of pandemic preparation strategies were implemented in different phases of the influenza pandemic. Management was carried out throughout the four phases.

### Phase of virus import blockage (late April to late June, 2009)

Influenza A (H1N1-2009) cases were not detected through general sentinel surveillance. Expansion of the monitoring of influenza activities was implemented in this phase. Strict management was made to block and delay the entry of pandemic virus into Zhanjiang. Thermal scanners were installed at airports and railway stations to detect travellers with fevers, especially in those travelled from South America and Pearl River Delta Areas. All asymptomatic contacts of patients with suspected and confirmed H1N1-2009 cases were quarantined at home. Symptomatic persons from an affected area were sent to hospitals, and nasopharyngeal swabs were collected for investigation. Main cases were handled immediately, samples were tested and cases were reported within 24 hours.

### Phase of limited transmission (early July to late August, 2009)

The first imported case of H1N1-2009 was detected during this period among a group of pupils returned from Guangzhou summer camp. Strict infection control measures were used to prevent further transmission, including identification, isolation, testing and antiviral treatment of every case, and every contact of suspected or confirmed cases was actively followed-up. A total of 11 H1N1 cases were reported during this period.

### Phase of local transmission (September 2009 to January 2010)

The monitoring of virus circulation in the population has been strengthened during this period. Active influenza case-finding was replaced by reporting and testing of patients with severe influenza-like illnesses or with other medically risky conditions. Six severe H1N1-2009 cases were detected and treated. All patients have recovered and no death was reported. A total of 36 outbreaks have been reported during this phase, and most outbreaks (86.1%) occurred in schools. Control measures have been implemented as soon as the outbreaks were reported. Oseltamivir chemoprophylaxis and other interventions, including school closures, isolation and oseltamivir treatment of confirmed cases, and home quarantine of close contacts of suspected or confirmed cases, were all implemented during school outbreaks. Thermal scanners that ran twice per day were installed at schools to detect fevers among students. Starting on October 12, free vaccination for H1N1-2009 virus was provided for medical staff members, students, teachers, and military men. Health education on the prevention and control of pandemic influenza A (H1N1-2009) was carried out both in schools and communities.

### Phase of epidemic subsidence (early February to late April 2010)

As of early February, the outbreak passed through the peak and has gradually subsided. Only five sporadic H1N1 cases were reported. The last H1N1 case was detected through general sentinel surveillance routine, and no new case was reported since then.

## Discussion

Our finding indicates that the vast majority (75.4%) of confirmed cases occurred in persons aged below 20 years. Schools were the main outbreak sites of pandemic influenza A (H1N1-2009) in Zhangjiang. It is reasonable to assume that the H1N1-2009 virus has been circulated in school-aged children. The outbreak evolved in Zhanjiang is similar to the outbreak in other areas [7]. The high infection rate of H1N1-2009 virus among teenagers and children suggested that there was no immunity to this antigenically novel influenza subtype. The lowest rate of infection was reported in patients over 60 years of age. This finding suggests that this group of people may have been previously exposed to a seasonal influenza A virus that is more closely related to the pandemic H1N1-2009 virus than to other influenza viruses, and that their sera might contain cross-reactive antibodies responding to the pandemic influenza A (H1N1-2009) virus [8].

Zhanjiang is located 700 kilometers southwest of Guangdong Province. The main city is surrounded by the sea, which resides in the tropical zone. Urban transportation mainly depends on road. Because of the relative inconvenient traffic network, it was expected that the entrance of the H1N1-2009 virus to Zhanjiang would be delayed than to other cities in Guangdong Province. Actually, the first case of H1N1-2009 was confirmed on May in Guangdong Province [9]. Two months later, the first imported H1N1-2009 case (also the first case) was reported in Zhanjiang on July 16. The H1N1-2009 virus was carried by a group of pupils who were on a summer camp trip held in Guangzhou. An outbreak of the influenza A (H1N1-2009) was emerged in this summer camp, and the influenza A (H1N1-2009) virus was disseminated into Zhanjiang and other cities. At the same time, several outbreaks of the influenza A (H1N1-2009) occurred in the Pearl River Delta Area [10,11].

Surveillance of the disease outbreaks in Zhanjiang identified an active period of seasonal influenza from March to September. Many cases of influenza A (H1N1-2009) were confirmed in September. In 2009, seasonal influenza peaked from March to July, and was the least active in November and December in Zhanjiang, which was different from previous years [12]. The co-circulation of seasonal influenza H3N2 and H1N1-2009 viruses suggested that seasonal influenza A and the H1N1-2009 viruses may have similar transmission dynamics. Cowling et al. [13] compared the spread of seasonal influenza A (H1N1), influenza A (H3N2), and H1N1-2009 viruses in Hong Kong, and found that the spread pattern of the H1N1-2009 virus was similar to that of influenza A viruses.

The epidemiologic pattern of the H1N1-2009 virus in Zhanjiang is similar to those in the Americas and Europe [14,15]; however, the occurrence of the peak pandemic waves of the H1N1-2009 virus in these areas differed in time. The peak wave of the H1N1-2009 virus arrived about the same time in Zhanjiang and some other tropical zones. Our results indicate that the H1N1-2009 virus dominated in November and December over seasonal influenza. As reported [16–18], during the disease outbreak, the peak of the epidemic was delayed to 2010 in some tropical zones of Asia.

The response strategies in Zhanjiang were favorable throughout the epidemic phases. Many patients were identified quickly. Management of the pandemic was carried out throughout the four phases, including both pharmaceutical interventions (the use of antiviral agents) and non-pharmaceutical interventions (e.g., thermal scanners, active case-finding, school closures, isolation, and quarantine). Our finding showed that the use of strict infection-control measures to prevent further transmission might have been useful in the initial phase of the outbreak. To achieve a maximum benefit, the control interventions should be changed according to the epidemic phases of the H1N1-2009 virus [19].

Laboratory diagnosis of influenza was carried out by virus isolation previously [12]. RT-PCR method has been introduced for virus detection as soon as the monitoring of H1N1-2009 was implemented in June of 2009. The sensitivity and accuracy of the RT-PCR detection method enabled an accurate measure of the real-time epidemic and progress of influenza activities. In the late epidemic period, Zhanjiang CDC provided H1N1-2009 vaccination for a variety of groups of people. Thus active surveillance and prevention are important aspects of an effective public health response, and influenza vaccination is the primary prevention tool [20].

Although we are in the post pandemic period [21], the H1N1-2009 virus is expected to circulate in a pattern similar to seasonal influenza, and will continue endangering younger population. It is now for us to answer the questions raised during the H1N1-2009 pandemic and to ponder how to prevent and respond in future public health emergencies.

## Conclusions

This study highlighted that adolescents were the major population struck by the H1N1-2009 virus. We confirmed that schools were the main outbreak sites of pandemic influenza A (H1N1-2009) in Zhangjiang, China. We also ascertained that the response strategies in Zhanjiang were favorable throughout the epidemic phases. In addition, we found that RT-PCR was an accurate and sensitive method for the detection and diagnosis of influenza A (H1N1-2009).
